# Automated classification of liver fibrosis stages using ultrasound imaging

**DOI:** 10.1186/s12880-024-01209-4

**Published:** 2024-02-06

**Authors:** Hyun-Cheol Park, YunSang Joo, O-Joun Lee, Kunkyu Lee, Tai-Kyong Song, Chang Choi, Moon Hyung Choi, Changhan Yoon

**Affiliations:** 1 Division of Industrial Mathematics, National Institute for Mathematical Sciences, 70, Yuseong-daero, Yuseong-gu, 34047 Daejeon, Republic of Korea; 2 Department of Computer Engineering, Gachon University, 1342, Seongnam-daero, Sujeong-gu, 13120 Seongnam-si, Gyeonggi-do Republic of Korea; 3 Department of Artificial Intelligence, The Catholic University of Korea, 43, Jibong-ro, 14662 Bucheon-si, Gyeonggi-do Republic of Korea; 4 Department of Electronic Engineering, Sogang University, 35 Baekbeom-ro, 04107 Seoul, Republic of Korea; 5 Department of Radiology, College of Medicine, The Catholic University of Korea, 222 Banpo-daero, Seoul, Republic of Korea; 6 Department of Biomedical Engineering, Department of Nanoscience and Engineering, Inje University, Inje-ro 197, 50834 Gimhae, Gyeongnam Republic of Korea

**Keywords:** Deep convolutional neural network, Liver fibrosis, Ultrasound imaging

## Abstract

**Background:**

Ultrasound imaging is the most frequently performed for the patients with chronic hepatitis or liver cirrhosis. However, ultrasound imaging is highly operator dependent and interpretation of ultrasound images is subjective, thus well-trained radiologist is required for evaluation. Automated classification of liver fibrosis could alleviate the shortage of skilled radiologist especially in low-to-middle income countries. The purposed of this study is to evaluate deep convolutional neural networks (DCNNs) for classifying the degree of liver fibrosis according to the METAVIR score using US images.

**Methods:**

We used ultrasound (US) images from two tertiary university hospitals. A total of 7920 US images from 933 patients were used for training/validation of DCNNs. All patient were underwent liver biopsy or hepatectomy, and liver fibrosis was categorized based on pathology results using the METAVIR score. Five well-established DCNNs (VGGNet, ResNet, DenseNet, EfficientNet and ViT) was implemented to predict the METAVIR score. The performance of DCNNs for five-level (F0/F1/F2/F3/F4) classification was evaluated through area under the receiver operating characteristic curve (AUC) with 95% confidential interval, accuracy, sensitivity, specificity, positive and negative likelihood ratio.

**Results:**

Similar mean AUC values were achieved for five models; VGGNet (0.96), ResNet (0.96), DenseNet (0.95), EfficientNet (0.96), and ViT (0.95). The same mean accuracy (0.94) and specificity values (0.96) were yielded for all models. In terms of sensitivity, EffcientNet achieved highest mean value (0.85) while the other models produced slightly lower values range from 0.82 to 0.84.

**Conclusion:**

In this study, we demonstrated that DCNNs can classify the staging of liver fibrosis according to METAVIR score with high performance using conventional B-mode images. Among them, EfficientNET that have fewer parameters and computation cost produced highest performance. From the results, we believe that DCNNs based classification of liver fibrosis may allow fast and accurate diagnosis of liver fibrosis without needs of additional equipment for add-on test and may be powerful tool for supporting radiologists in clinical practice.

## Introduction

Damage of hepatocytes caused by various etiologies such as infection, non-alcoholic fatty liver, alcohol, inherited metabolic disease, immune disease, and drug induces activation of hepatic stellate cell, secretion of cytokines and accumulation of collagens, resulting in liver fibrosis [1]. Cirrhosis is the most severe and irreversible stage of liver fibrosis, which can progress to portal hypertension and hepatocellular carcinoma [2]. Thus, accurate diagnosis of liver fibrosis in early stage is of great importance in clinical practice since prognosis and management of chronic liver diseases are related to severity of liver fibrosis.

The histopathological examination through liver biopsy is the gold standard for liver fibrosis diagnosis and staging. However, liver biopsy is prone to sampling errors due to examination of small liver parenchyma specimen, and to intra-/inter-observer variations [3, 4]. In addition, it is invasive that can cause various complications and may lead to death. Thus, repeated liver biopsy to trace disease progression is not recommended.

**Fig. 1:**
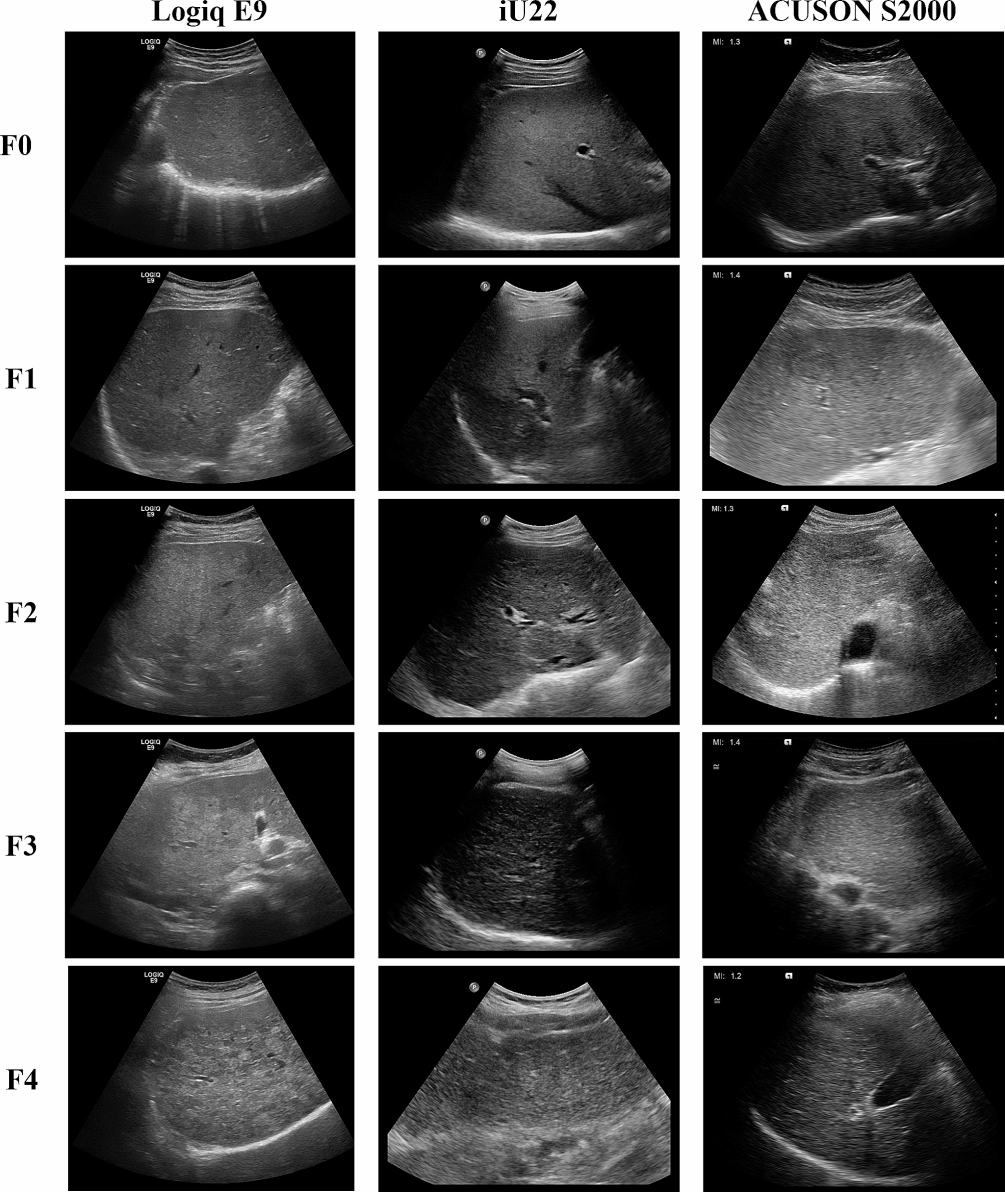
Representative US images of liver fibrosis. Alteration of parenchymal echogenicity (graded as fine echotexture, mildly coarse and highly coarse) and surface nodularity can be identified as the liver fibrosis progress

To overcome these limitation, non-invasive methods such as magnetic resonance imaging (MRI), computed tomography (CT) and ultrasound (US) imaging for accessing liver fibrosis have been investigated and shown promising results despite the need for additional time and equipment [5–7]. These imaging modalities provide not only morphological information (e.g., parenchymal changes and portal hypertension) but also functional information (e.g., stiffness of tissue) that is related to the stage of fibrosis. Among these, US imaging is the widely available modality with no ionizing radiation. Thus, US imaging is the most frequently performed in the regular follow-up of patients with chronic hepatitis or liver cirrhosis for the detection of hepatocellular carcinoma and an evaluation of the degree of liver fibrosis. It was reported that the progression of fibrosis include alteration of parenchymal echogenicity (graded as fine echotexture, mildly coarse and highly coarse) and surface nodularity [8]. Figure 1 shows the representative US images of liver fibrosis for each stage. Although these imaging findings have a correlation with the degree of liver fibrosis, interpreting the findings is subjective thus well-trained radiologist is required for evaluation.

Recently, several studies have shown that deep convolutional neural networks (DCNNs) based diagnosis and assessment of liver fibrosis is viable solution using MR and CT images [9–13]. A DCNN-based quaternary classification model was developed to classify liver cirrhosis (F0/F1/F23/F4) using US B-mode images [14]. In the method, VGGNet was applied for transfer learning and the accuracy of VGGNet for METAVIR score classification was 83.5%. However, the developed automated classification model was trained using images obtained by US machines from three major vendors (i.e., GE healthcare, Philips Medical Systems and Siemens Medical Solution). Because the model learned from images acquired from a limited domain would be biased toward the characteristic of the corresponding machine, it may achieve a weak performance when applying US images acquired from another domain. Considering that there are many different types of US machines, multi-domain data are necessary to reflect real clinical situations.

The purpose of this study was to access the performances of popular and well-established DCNNs (VGGNet, ResNet, DenseNet, EfficientNet and ViT) to identify that which of DCNNs trained on the ImageNet dataset will perform best for the classification of liver fibrosis using US images obtained from 11 different US machine.

### Related works

In this section, we describe previous works on US image classification using deep learning. It was reported that an accuracy of 90.6% in identifying fatty liver disease from US images could be achieved by using the VGG-16 model [15]. A novel multi-task learning approach for segmenting and classifying tumors in breast ultrasound images was proposed [16]. In the method, they used VNet as the backbone network. The proposed network comprised an encoder-decoder network for segmentation and a lightweight multi-scale network was integrated for classification. A regularized spatial transformer network was proposed for automated pleural effusion detection in lung US and an accuracy of 91.12% was achieved in classification of pleural effusion [17]. The performance of ResNet pre-trained with the ImageNet dataset for classification of chronic liver disease in renal US imaging was evaluated [18]. For classifying thyroid nodules and breast lesions in US images, TNet and BNet using pre-trained VGG-19 was developed and could achieve classification accuracies of 86.3% and 86.5%, repectively [19]. A deep learning architecture that includes a feature extraction network, an attention-based feature aggregation network, and a classification network was also proposed for diagnosing thyroid nodules [20].

## Materials and methods

### Ethics committee approval

US images from two tertiary university hospitals (Seoul St. Mary’s Hospital, The Catholic University of Korea and Eunpyeong St. Mary’s Hospital, The Catholic University of Korea) were used for the training and validation. This study was approved by the institutional review boards of both hospitals (Seoul St. Mary’s Hospital: KC20RISI0869 and Eunpyeong St. Mary’s Hospital: PC20RISI0229). The requirement for informed consent was waived because of the retrospective study design.

### Training and validation dataset

Table 1 summarizes the clinical characteristics of the 933 patients (556 male patients) included in this study. The median age was 54 years old (interquartile range, 44–63). The numbers of patients and images that were used in this study were summarized according to US machines in Table 2. Only patients who underwent liver biopsy or hepatectomy between 2011 and 2020 at the Seoul St. Mary’s Hospital, or between 2019 and 2020 at the Eunpyeong St. Mary’s Hospital, were eligible for this study. Although non-invasive methods such as transient elastography are widely used to evaluate liver fibrosis, there is a possibility that errors will occur in cases of a contracted liver or ascites. Among them, patients who underwent a liver US within 3 months prior to biopsy or surgery were included in this study, with 745 patients from the Seoul St. Mary’s Hospital and 188 patients from the Eunpyeong St. Mary’s Hospital in the training/validation. A radiologist with 11 years of experience with abdominal US reviewed all images and selected liver images regardless of scanning plane. All images obtained with using a convex probe. In this study, for the automated diagnosis of liver fibrosis, we categorized liver fibrosis based on pathology results of biopsy or hepatectomy using the METAVIR score [21]. Note that a pathology report is a medical document that provides final diagnosis based on microscopic examination of the tissue specimen. The METAVIR score consists of five classes, i.e., F0, F1, F2, F3, and F4. Here, F0 indicates no fibrosis; F1, portal fibrosis without septa and an insignificant abnormal area; F2, portal fibrosis with few septa and abnormalities in an area wider than with F1; F3, numerous septa without cirrhosis and prominent abnormalities; and F4, cirrhosis. We experimented with a five-level classification of F0, F1, F2, F3, and F4 for liver fibrosis.

**Table 1:** The characteristics of patients in the data

	Values
Number of patients (n)	933
Number of images (n)	7920
Sex (M:F)	556: 377
Age (years)	54 (44–63)
Total bilirubin (mg/dL)	0.88 (0.63–1.54)
AST (U/L)	45 (27–98)
ALT (U/L)	41 (23–100)
Albumin (g/dL)	4.1 (3.5–4.4)
Platelet count (10^9^/L)	174 (110–235)
Prothrombin time (INR)	1.09 (1.03–1.20)
METAVIR score	
F0	262 (28.1%)
F1	124 (13.3%)
F2	103 (11.0%)
F3	134 (14.4%)
F4	310 (33.2%)
Number of ultrasound machines	11

AST, aspartate transaminase; ALT, alanine transaminase; INR, international normalized ratio

**Table 2:** Distribution of liver fibrosis stages in the training and validation data

	Machine model	Manufacturer	Number of patients	Year of manufacture	Fibrosis				
					F0	F1	F2	F3	F4
**Training** **/** **Validation**	SSD-5000	Aloka	13	2005	2	0	1	1	9
	EUB-7500	Hitachi	169	2009	31	27	27	29	55
	IU22	Philips	175	2009	86	13	7	17	52
	Logiq E9	GE	108	2013	46	4	6	17	35
	Prosound F75	Hitachi	107	2016	17	21	28	11	30
	S2000	Siemens	105	2009	18	8	6	13	60
	Sequoia	Acuson	59	2009	7	2	4	7	39
	Epiq	Philips	11	2019	1	5	0	2	3
	Logiq E10	GE	126	2019	38	28	11	27	22
	Logiq S8	GE	50	2019	13	10	11	8	9
	Aplio 500	Toshiba	10	2011	1	3	2	2	2

### Data preprocessing

The distribution of the grades of liver fibrosis is shown in Table 1. Training and validation data were used at a ratio of 8:2 for the entire dataset. Before training a model, the distribution ratio of the dataset must be considered. In particular, data on diseases that are difficult to detect in early stage, such as liver fibrosis, have an imbalance in terms of degree. In general, F0 is easily obtained, and such data occupy 28.1% of the dataset. F4, the end stage of liver fibrosis, accounted for 33.2% of the dataset. However, the proportions of F1 (13.3%), F2 (11.0%), and F3 (14.4%) were relatively small because only a few patients were examined during the early stages of liver fibrosis. Such data imbalance can bias and overfit the model training [22, 23]. To solve this problem, data augmentation should be conducted [24]. Using a computer vision method, a data augmentation of the images expands the size of a limited dataset. In general, flipping, color jitter, cropping, rotation, translation, and noise generation are used for such augmentation [25]. However, data augmentation may undermine the inherent meaning of the original data depending on the augmentation method applied. Since the US images using a convex array are fan shape, horizontal flips were only applied for data augmentation in this work. The final images were normalized and resized to a pixel resolution of 224 × 224 for model training. The size of input images should be adjusted appropriately to align with the dimensions permitted by the DCNN models. The approved input size for the key models used in our experiments is 224 × 224, and it is recommended to maintain a standardized resolution for objective experimentation. Furthermore, we employ transfer learning using pre-trained parameters from ImageNet. To optimize the effectiveness of transfer learning, it is crucial to minimize alterations to the model.

### Implementation of DCNNs

The models were trained using VGGNet-16, ResNet-50, DenseNet-121, EfficientNet-B0, and ViT [26–30]. Each model commonly consists of an encoder f*θ* and a classifier g*θ*. The encoder f*θ* extracts mid-level features through a convolution, and the classifier g*θ* is a linear classifier that classifies the final features. The encoder *f*_*θ*_ follows the architectures of the VGG, ResNet, DenseNet, EfficientNet, and ViT models. Notably, each model exhibits distinctive implementation features. VGG is characterized by its simplicity and uniformity. With 16 weight layers, VGG employs small 3 × 3 convolutional filters and max-pooling layers. ResNet utilizes residual blocks with skip connections, addressing the vanishing gradient problem by enabling the flow of gradients through the network. DenseNet redefines connectivity in neural networks. DenseNet’s dense blocks connect all layers by concatenating feature maps, promoting maximal information flow. EfficientNet excels in balancing model depth, width, and resolution. Its compound scaling method adjusts these dimensions simultaneously, achieving state-of-the-art performance with a smaller model. EfficientNet represents an innovative approach to optimizing computational efficiency. ViT (Vision Transformer) introduced the transformer architecture to computer vision. Departing from traditional convolutional structures, ViT tokenizes input images into patches and employs self-attention mechanisms. This pioneering approach allows ViT to capture global dependencies effectively, achieving performance comparable to or surpassing traditional convolutional models at a fraction of the computational cost.

The final classifier *g* is implemented as a fully-connected layer, constituting a linear classifier. The output value of *g* is normalized to a probability using the softmax function. The objective cross-entropy function is configured such that the probability of the target class is maximized. Finally, the parameter *θ* is trained to optimize the objective function.

**Fig. 2:**
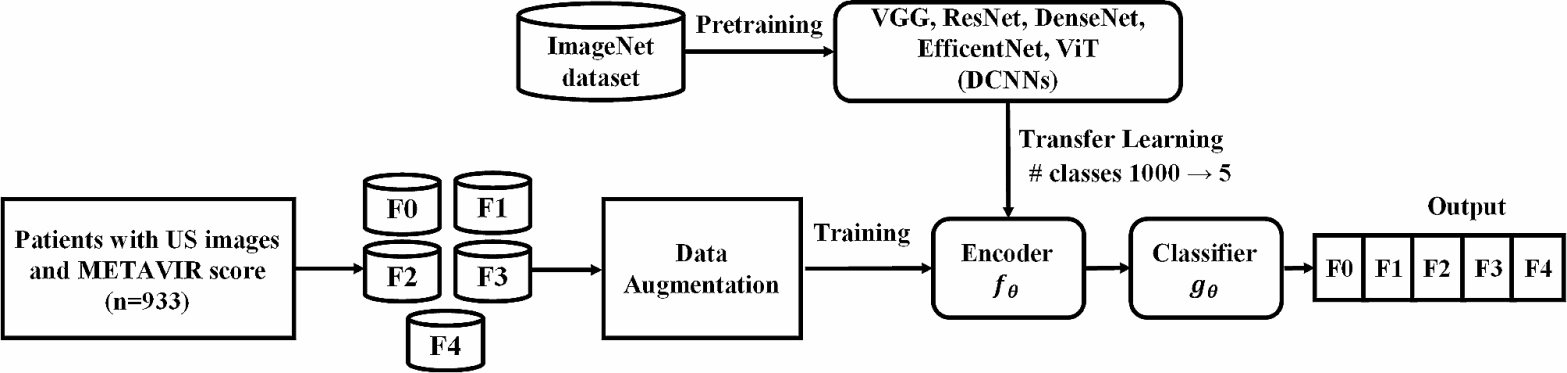
Training diagram of DCNNs. Five models (VGG16, ResNet50, DenseNet121, EfficientNet-B7, and ViT) were trained using US images from 11 different machines. The number of data is based on patients. DCNN, deep convolutional neural network; US, ultrasound

We applied transfer learning for model training (Fig. 2) because scratch learning is valid when the number of training data is more than 5000 per class [31, 32]. Transfer learning uses a model trained on an extensive dataset from another domain. In general, the ImageNet dataset, which consists of 1000 classes, is widely used for pre-training. Model training using extensive datasets is suitable for extracting meaningful features from input images. Because the pre-trained model has been trained to find high-level features, the convolution filter of the model is better optimized than scratch learning when learning a new domain from the pre-training. If the pre-trained and post-trained datasets are in similar domains, the models can yield valid results even when freezing the convolution layers. However, in post-training using medical images, the model must be retrained based on the overall parameters because ImageNet and medical images have different cardinal features. In this study, after transfer learning on ImageNet, we conducted fine-tuned the model using US images [33].

In this study, the loss function for model training was CrossEntropyLoss by Negative Loglikelihood, and the optimization algorithm and learning-rate scheduler were the Adam optimizer and CosineAnnealingLR, respectively. The initial learning rate started at 0.0001 and was adjusted to a value close to zero every 50th epoch by the scheduler. We trained the model for 1000 epochs using a batch size of 64.

### Evaluation metrics

The performance of DCNNs was evaluated through accuracy, sensitivity, specificity, positive and negative likelihood ratio. In addition, area under the receiver operating characteristic curve (AUC) with 95% confidential interval for five-level (F0/F1/F2/F3/F4) classification was used to assess the efficiency of DCNNs.

## Results

**Fig. 3:**
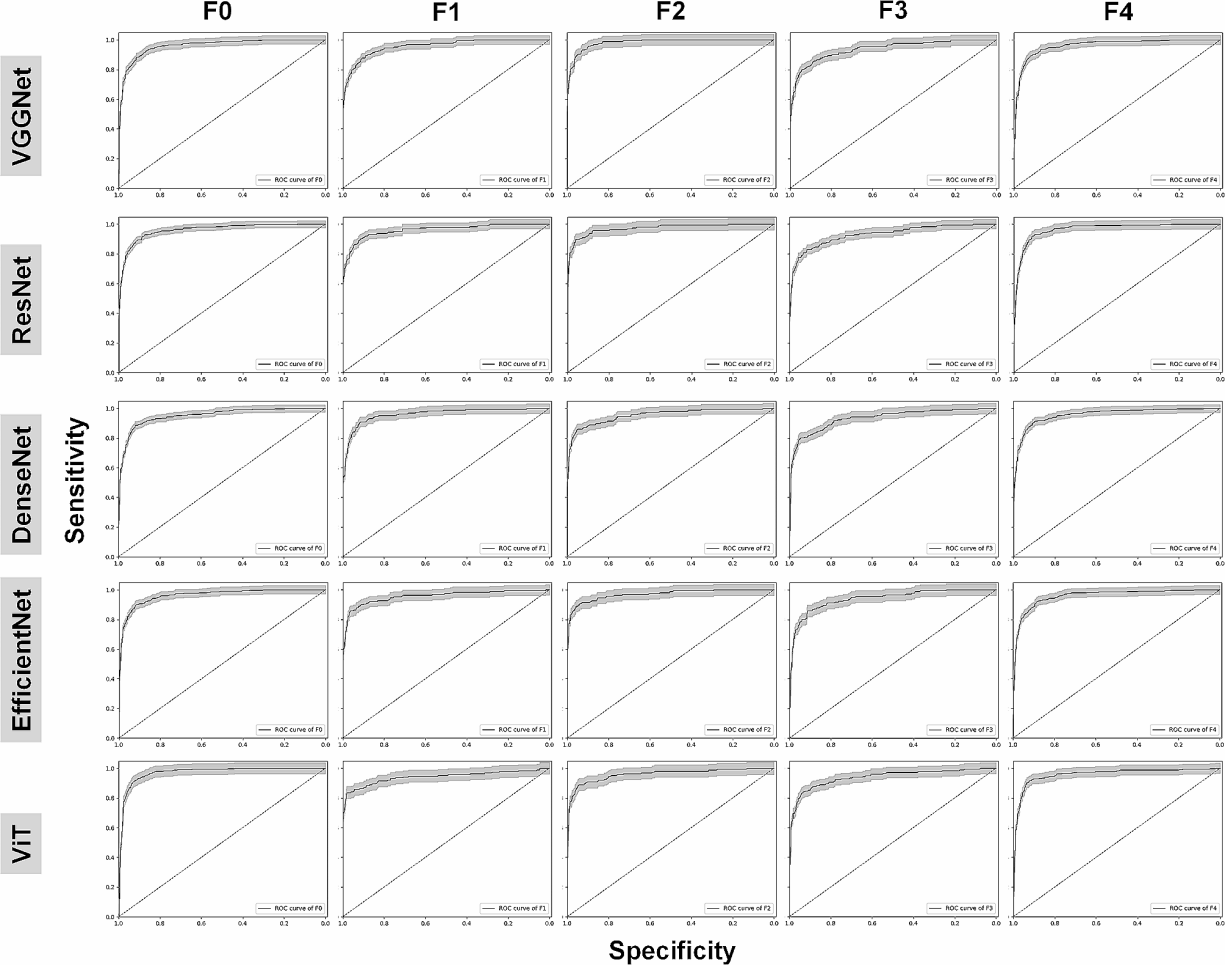
Receive operation characteristic curves with 95% confidence intervals for classification of liver fibrosis according to METAVIR score using VGGNet, ResNet, DenseNet, EfficientNet and ViT, respectively

Table 3 summarizes the diagnostic performance of DCNNs for five-level classification. Similar AUC values were achieved for five models (Fig. 3); VGGNet (mean: 0.96, range: 0.94–0.98), ResNet (0.96, 0.93–0.97), DenseNet (0.95, 0.94–0.96), EfficientNet (0.96, 0.94–0.97), and ViT (0.95, 0.94–0.97). The same mean accuracy value (0.94) was yielded for all models. In terms of sensitivity, EffcientNet achieved highest value (0.85, 0.80–0.89) while the other models produced slightly lower values; VGGNet (0.82, 0.72–0.89), ResNet (0.84, 0.75–0.90), DenseNet (0.82, 0.75–0.89), and ViT (0.83, 0.76–0.92). All model achieved approximately the same mean specificity value (0.96).

**Table 3:** Diagnostic performance of DCNNs for five-level classification

Model	Staging	AUC	Accuracy	Sensitivity	Specificity	PLR	NLR
**VGGnet**	F0	0.96	0.91	0.89	0.92	11.44	0.12
	F1	0.96	0.94	0.72	0.98	31.35	0.29
	F2	0.98	0.96	0.86	0.98	41	0.14
	F3	0.94	0.93	0.74	0.97	21.2	0.27
	F4	0.96	0.92	0.87	0.94	15.05	0.13
**Resnet**	F0	0.96	0.93	0.9	0.94	15.24	0.11
	F1	0.96	0.96	0.79	0.99	53	0.21
	F2	0.97	0.96	0.84	0.98	44.32	0.16
	F3	0.93	0.94	0.75	0.97	22.15	0.26
	F4	0.97	0.92	0.89	0.94	13.68	0.12
**DenseNet**	F0	0.95	0.91	0.89	0.93	12.19	0.12
	F1	0.96	0.96	0.81	0.98	47.35	0.2
	F2	0.95	0.95	0.82	0.97	28.21	0.19
	F3	0.94	0.93	0.75	0.96	20.92	0.26
	F4	0.96	0.92	0.86	0.95	16.46	0.15
**EfficientNet**	F0	0.96	0.93	0.89	0.95	18.58	0.11
	F1	0.96	0.95	0.79	0.98	39.75	0.21
	F2	0.97	0.96	0.87	0.98	34.92	0.13
	F3	0.94	0.94	0.81	0.96	18.48	0.2
	F4	0.96	0.93	0.86	0.95	17.83	0.15
**ViT**	F0	0.97	0.92	0.91	0.93	13.07	0.09
	F1	0.94	0.95	0.77	0.99	51.6	0.23
	F2	0.96	0.95	0.82	0.97	26.58	0.18
	F3	0.94	0.93	0.76	0.96	20.11	0.25
	F4	0.96	0.93	0.86	0.96	19.52	0.15

## Discussion

In this study, we demonstrated that DCNNs trained by transfer learning on ImageNet can classify the staging of liver fibrosis according to METAVIR score with high performance (AUC: > 0.95, accuracy: 0.94) using conventional B-mode images from multiple US machines. Five different DCNNs showed good diagnostic performance, and the highest performance was achieved with comparably less computational complex network, i.e., EfficientNET.

Recently, various studies have been conducted on DCNN-based automatic detection and classification using US images [34]. Automated staging of liver fibrosis based on US images was also investigated [14]. Although high performance (AUC: 0.90, accuracy: 0.94) was achieved for classification of significant fibrosis (F2 or greater), the accuracy for quadrant classification (F0/F1/F23/F4) was relatively low (0.83). This is mainly due to the imbalance of training dataset. In our method, we conducted data augmentation to balance data distribution, which prevent bias and overfit the model training. In addition, our result showed that computational complex networks do not always guarantee better performance. The use of DCNNs with less computations have several advantage since it can lower hardware complexity and reduce training time. This will allow fast and easy implementation of DCNNs for automated classification on conventional US machines.

US is commonly used to evaluate the liver in patients with chronic liver disease. Liver fibrosis stage is difficult to predict solely based on US B-mode images even with regular follow-up because the morphology or echogenicity of the liver does not change remarkably in the early stage of liver fibrosis. Therefore, US elastography has been used as a promising imaging technique to evaluate the elastic modulus of tissues and to evaluate liver fibrosis [35–37]. However, liver fibrosis stage was divided into two groups, such as F4 versus others, in most studies since it is still difficult to classify five stages of liver fibrosis even with elastography. Once our approach is mounted on existing machines, it will be a convenient alternative tool for assessing liver fibrosis without selection of scanning plane and needs of additional equipment for add-on test such as Fibroscan and elastography, especially in low-to-middle income countries.

In this work, we compared the results of the main backbone models ranging from shallow to deep networks. While various state-of-the-art (SOTA) models exist, the majority of them adopt derivative structures from our experimental models. Therefore, for an objective assessment of the effectiveness of DCNN, it is appropriate to evaluate the performance using the fundamental forms that constitute the backbone, including the latest baseline such as Vit.

Our study has several limitations. First, since we used the data augmentation to balance the dataset for each stage, it could undermine the performance of networks. For our approach to be used in practice, the model should be trained using sufficiently large dataset (more than 5000 case for each stage) without data augmentation. Second, although we included as many US machines (11 different machines) as possible, there are dozens of companies that manufacture US scanners. Since all manufacturers have their own image processing methods such as filtering and speckle reduction, echotexture or feature of images is different from each other (see Fig. 1). For versatile solution, we may need to include images from every US machines for training. Otherwise, model need to be trained individually by using images from each US machine. Third, the US images used for training were acquired by well-trained radiologists. Considering that US is highly operator-dependent, images from under-trained radiologists may need to be included for low-to-middle income countries with weak health care systems. Fourth, due to the computational burden, we resized images to 224 × 224 for model training, thus our approach may use the overall morphological features such as liver surface irregularity to classify liver fibrosis. The alteration of parenchymal echogenicity also convey useful information to predict liver fibrosis [8]. Thus, if we can use whole B-mode image without sacrificing the resolution, the performance of model could be improved. Finally, due to the nature of retrospective study, we could not include information regarding hepatitis B or C and alcohol consumption. However, liver fibrosis is diagnosed regardless of the cause, thus absence of etiology would not undermine our experiment results.

In conclusion, we have demonstrated that DCNNs can classify METAVIR score using conventional US images with high accuracy. Given the fact that US imaging is widely available modality and the most frequently used in the regular follow-up of patient with chronic liver disease, DCNNs based classification of liver fibrosis using B-mode images will be powerful tool for supporting radiologists in clinical practice, which however need further improvement and validation would be required.

## Data Availability

The datasets used and/or analyzed during the current study available from the corresponding author on reasonable request.
